# The trochanteric double contour is a valuable landmark for assessing femoral offset underestimation on standard radiographs: a retrospective study

**DOI:** 10.1186/s12891-021-04133-8

**Published:** 2021-03-29

**Authors:** Stefan Blümel, Vincent A. Stadelmann, Marco Brioschi, Alexander Küffer, Michael Leunig, Hannes A. Rüdiger

**Affiliations:** 1 Department of Hip and Knee Surgery, Schulthess Klinik, Zürich, Switzerland; 2 Department of Teaching, Research and Development, Schulthess Klinik, Lengghalde 2, CH-8008 Zürich, Switzerland; 3 Department of Neurosurgery, Schulthess Klinik, Zürich, Switzerland

**Keywords:** Total hip arthroplasty, Femoral offset, Templating, Projection error, Double contour, Trochanter major

## Abstract

**Background:**

Inaccurate projection on standard pelvic radiographs leads to the underestimation of femoral offset—a critical determinant of postoperative hip function—during total hip arthroplasty (THA) templating. We noted that the posteromedial facet of the greater trochanter and piriformis fossa form a double contour on radiographs, which may be valuable in determining the risk of underestimating femoral offset. We evaluate whether projection errors can be predicted based on the double contour width.

**Methods:**

Plain anteroposterior (AP) pelvic radiographs and magnetic resonance images (MRIs) of 64 adult hips were evaluated retrospectively. Apparent femoral offset, apparent femoral head diameter and double contour widths were evaluated from the radiographs. X-ray projection errors were estimated by comparison to the true neck length measured on MRIs after calibration to the femoral heads. Multivariate analysis with backward elimination was used to detect associations between the double contour width and radiographic projection errors. Femoral offset underestimation below 10% was considered acceptable for templating.

**Results:**

The narrowest width of the double line between the femoral neck and piriformis fossa is significantly associated with projection error. When double line widths exceed 5 mm, the risk of projection error greater than 10% is significantly increased compared to narrower double lines, and the acceptability rate for templating drops below 80% (*p* = 0.02).

**Conclusion:**

The double contour width is a potential landmark for excluding pelvic AP radiographs unsuitable for THA templating due to inaccurate femoral rotation.

## Background

The reconstruction of abductor lever arms is critical for hip function after total hip arthroplasty (THA). In clinical practice, femoral offset—defined as the distance between the center of the femoral head and anatomical axis of the proximal femur—is used to simplify the biomechanical analysis of native or prosthetic hip joints and is an important determinant of postoperative hip function [1]. Postoperative changes in femoral offset may lead to altered muscular function that can result in hip abductor muscle insufficiency and pain as well as early prosthesis wear [2, 3]. Furthermore, loss of femoral offset necessitates increased abductor muscle force to maintain normal gait pattern [4], and a loss exceeding 15% can result in gait alterations [5].

Assessment of femoral offset is routinely made on plain anteroposterior (AP) radiographs of the pelvis. However, this measurement is regularly underestimated because of incorrect projection. True anatomical hip measurements are made using computed tomography (CT), but this technique is limited in routine clinical work because of the associated radiation exposure levels and high costs. When compared to CT scan measurements, radiographic femoral offset is critically underestimated in as many as 28% of THA patients [6].

For templating THA on plain radiographs, it is pivotal to assess the projection quality resulting from possible femur malrotation. The lesser trochanter is commonly used as a guide for assessing femoral anteversion, but quantifying rotation with it is imprecise [7, 8]. Another possible landmark to consider is the external obturator footprint: together with the greater trochanter posteromedial facet, they are visible as two contours on AP radiographs [9]. If this easily identifiable double contour is narrow and almost superimposed, the underestimation of femoral offset appears to be minimal. Therefore, our aim was to examine whether the double contour width is predictive of the projection error on plain pelvic AP radiographs. We hypothesized that femoral rotation relative to the x-ray plane may be estimated by measuring the distance between the two contours of the posteromedial trochanteric facet, which may be considered a good predictor of femoral projection.

## Methods

For this observational study, we retrospectively examined radiographs and magnetic resonance images (MRIs) from patients who were aged 16 years and older and consulted our institution for any hip condition between January 2014 until December 2018. This analysis was approved by the Cantonal Ethics Committee of Zurich (KEK-ZH-Nr. 2015–0258) in conjunction with the 1964 Helsinki Declaration and its later amendments or comparable ethical standards.

We included patients who had pelvic AP radiographs and hip joint MRI with femoral antetorsion view, acquired within 1 year of each other. Of 72 patients with complete image documentation, eight were excluded for the following reasons: open growth plates (*n* = 1), poor image quality (*n* = 1), severe hip dysplasia (*n* = 2), Morbus Perthes (*n* = 2) or a prior hip surgery (*n* = 2). Our final cohort included 64 patients with a mean age of 30 years (Table 1). All patients provided written informed consent to use their clinical data for research and publication purposes.

**Table 1:** Patient data

	Data^*^
Patients/hips	64/64
Age (years)	29.9 ± 11.1 (16–77)
Sex (male/female)	26/38
Side (right/left)	37/27
Time between MRI and radiograph (days)	15 ± 75 (− 186–197)

*Values presented either as the number or mean ± standard deviation (range)

### Hip image evaluations

**Fig. 1:**
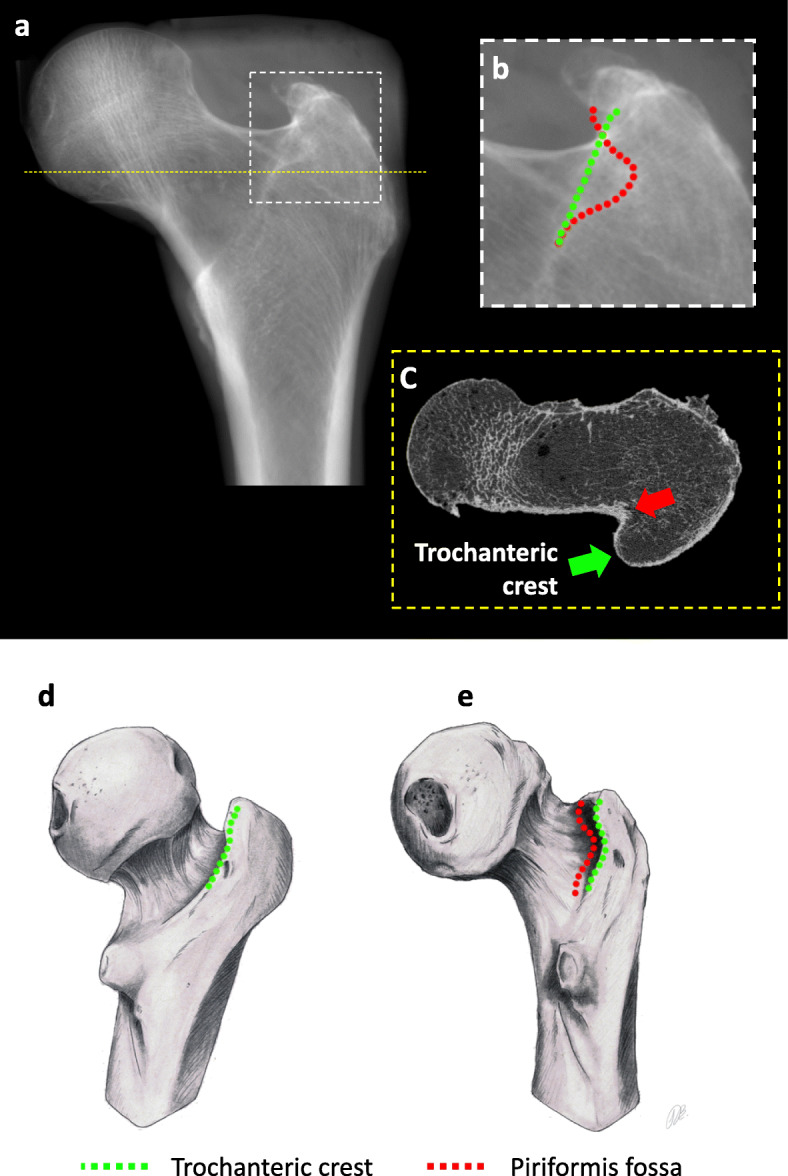
The double contour highlighted within the boxed area (**a**) on the pelvic AP radiograph comprises the most common aspect of one S-shaped line (outlined by red dots) and the second line with minimal curvature (green dots) (**b**). On a high-resolution CT scan (**c**), the origin of the contours are identified as the trochanteric crest (green arrow) and piriformis fossa (red arrow). In profile view, both lines are superimposed (**d**) and as the femur rotates, the two lines appear as separate elements (**e**)

Plain pelvic radiographs were acquired at 81 kV/40 mAs according to our clinical protocol with a slight internal rotation of the legs of 10–15° to compensate for the femoral antetorsion, and the radiographic cassette was placed two fingerbreadth over the iliac crest. On these radiographs, the double contour is the lateral part of the intertrochanteric crest at the posteromedial facet of the greater trochanter and projection of the convex cortex of the piriformis fossa (Fig. 1). We focused on measuring the double contour separation width and apparent femoral neck length. The latter was chosen because unlike femoral offset, femoral neck length is independent of femoral axis that is difficult to assess accurately due to the limited field on pelvic radiographs, CT scans and MRIs.

**Fig. 2:**
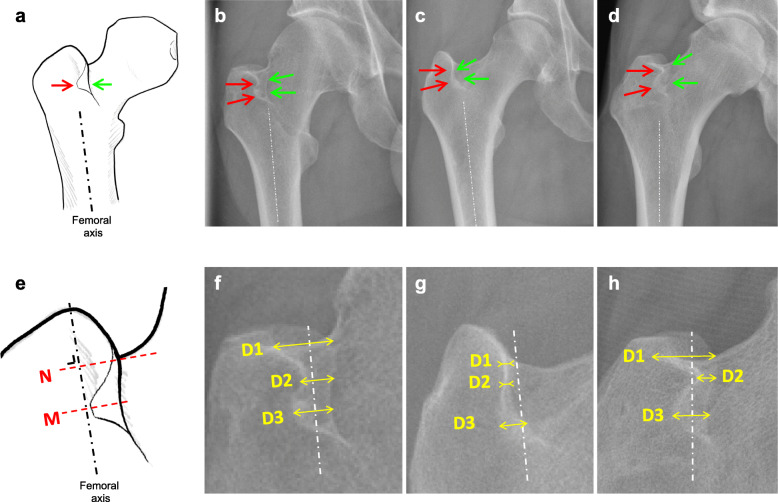
**a** The double contour lines appear on the diagrammatic representation of the femoral head with red and green arrows indicating their origins on the trochanteric crest and piriformis fossa, respectively. On standard radiographs, the classic anatomical variant (**b**) comprises one S-shaped (red arrows) and one less curved line (green arrows); (**c**) superimposed lines are less common; and (**d**) the last and more rare variant is seen as two curvy lines. **e** Width measurement was defined at three levels perpendicular to the femoral axis (black hatched line). The measurements are defined between the levels of the neck (N) and maximum double line width (M) (f-h), where D1 = femoral neck level, D2 = minimum thickness between D1 and D3, and D3 = maximum double line thickness

Multiple anatomical variants of the double contour can be observed. The most common variant appears as an S-shaped line and one less curved line, another less common appears as superimposed lines and the last, more rare variant, is seen as two curvy lines (Fig. 2a-d). To account for these variants, we defined the width of the double contour (including line thickness) at three levels perpendicular to the femoral axis: D1 at the femoral neck level, D3 as the maximum thickness of the double line and D2 as the minimum thickness between D1 and D3 (Fig. 2e-h).

**Fig. 3:**
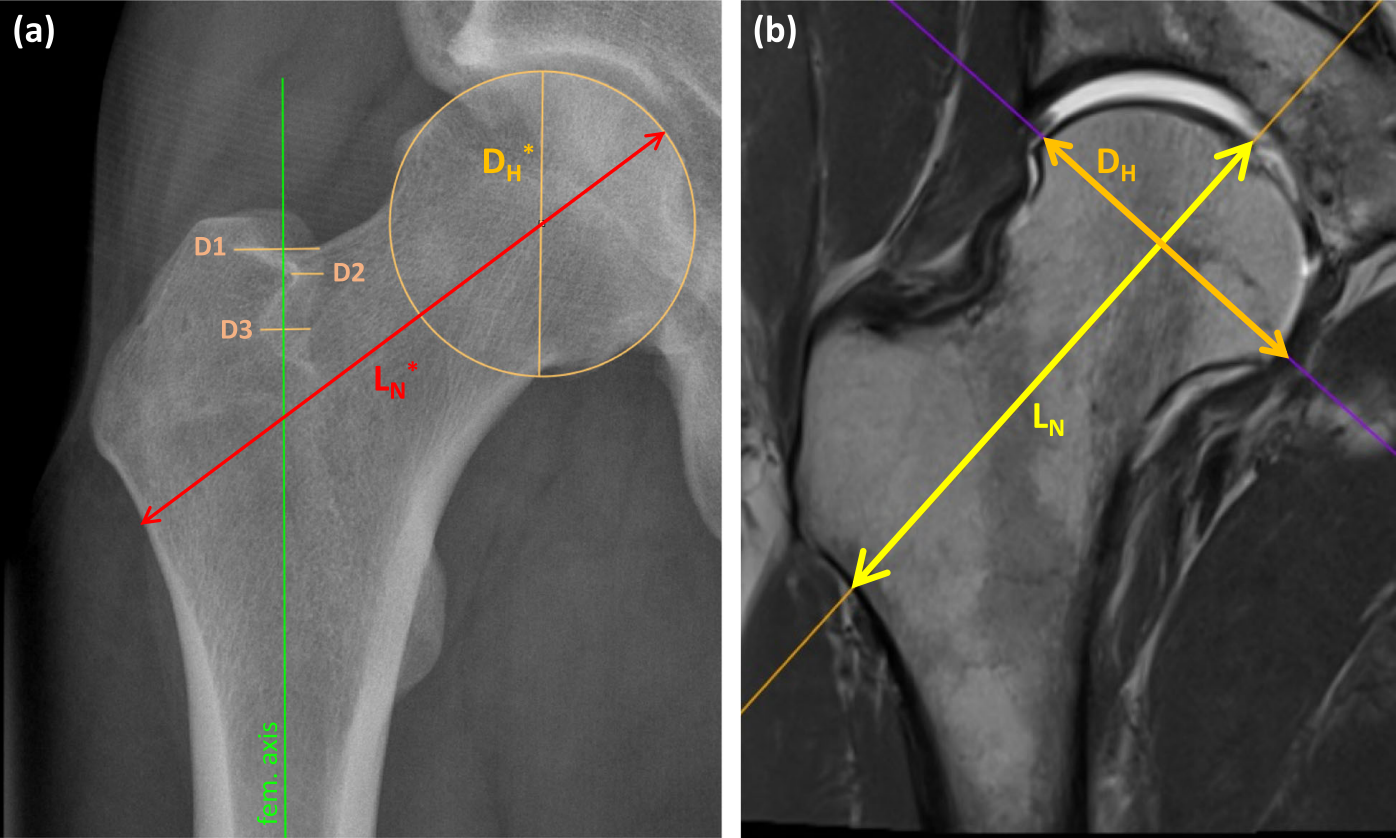
**a** The apparent femoral neck length, L_N_^*^, was measured on the anteroposterior radiograph as the full bone length spanning the cortical surface of the femoral head to the opposing cortical surface along the femoral neck axis. Apparent femoral head diameter (D_H_^*^) was measured by fitting a circle to the articular surface. **b** True femoral neck length and femoral head diameter were measured on the magnetic resonance image in the sagittal plane of the femur and compared to the corresponding radiographic measurements

For each pelvic AP radiograph, the double contour widths (D1, D2, D3), apparent femoral neck axis length (L_N_*) and apparent femoral head diameter (D_H_* for calibration) were measured manually with the measurement tools of the DICOM viewer (JiveX 5.0.2.2, VISUS Health IT GmbH, Bochum, Germany). For L_N_*, we measured the full bone length spanning the cortical surface of the femoral head to the opposing cortical surface along the femoral neck axis. D_H_* was measured by fitting a circle to the articular surface (Fig. 3a). To evaluate the inter-rater reliability of these measures, twenty randomly selected images were evaluated independently by three physicians. Intra-rater reliability was determined based on one rater reading the same images twice with 4 weeks between assessments.

MRIs of the hip joint with a view of the distal femur for antetorsion measurement were acquired as described by Sutter et al. [1]. True femoral neck axis length (L_N_) and true femoral head diameter (D_H_) were also measured on MRIs of each hip in our cohort to calculate the underestimation in femoral neck length projection on the corresponding plain radiographs. Using multiplanar reconstruction (3D MPR) in the DICOM viewer, the MRIs were manually aligned parallel to the plane through both the femoral neck axis and the axis through the proximal femoral diaphysis. D_H_ and L_N_ were then measured using the same landmarks as those observed on the plain radiographs (Fig. 3b). Femoral antetorsion was also quantified on MRIs as described by Botser et al. [10].

Two main factors affect length measurements on a radiograph: (1) geometric magnification resulting from the distance between the object (patient) and film (camera) and (2) geometric distortion (projection error) resulting from the angle between the object and film relative to the x-ray beam [9]. We used the femoral head diameter to correct for magnification with a correction factor (*D*_*H*_/*D*_*H*∗_): the apparent neck length corrected for magnification (*L*_*N*_*#)* was calculated as ,

$$ {L}_{N^{\#}}={L}_{N^{\ast }}\times {D}_H/{D}_{H^{\ast }} $$

Projection error, as indicated by *E*_*proj*_ was calculated from the difference between the apparent femoral neck length (corrected for magnification) and true femoral neck length,

$$ Eproj=\left({L}_N-{L}_{N^{\#}}\right)/{L}_N $$

### Statistical analysis

All analyses were carried out using R [11]. Normality of the data was confirmed with Q-Q plots and Shapiro-Wilk tests. The data was modelled with generalised multivariate regression analyses using backward elimination to determine whether there was an independent association between the calculated projection errors and anatomical parameters with the significance level set to *p* <  0.05. Intraclass correlation coefficients (ICCs) were calculated using the *irr* package [12] to determine the intra- and inter-rater reliabilities of all image measurements. In addition, double contour widths were categorised by intervals of 2 mm and an “acceptability rate” (AR) was defined as the fraction of radiographs with a projection error < 10% within a defined interval. Distributions of ARs were compared with Chi-square proportion tests. Study data were collected and managed using REDCap electronic data capture tools hosted at our clinic [13, 14].

## Results

The anatomical measurements of antetorsion, neck length, head diameter and double contour width were normally distributed for our patient cohort (Table 2); all measurements, except antetorsion, were significantly larger for males (*p* ≤ 0.0001). The mean calculated projection error was 2% higher for female patients (*p* = 0.024).

**Table 2:** Distribution of anatomical measurements made on plain pelvic radiographs according to sex

	Male		Female		***P***-value
	Mean ± SD	Range	Mean ± SD	Range	
Antetorsion (°)	8.0 ± 10.0	(−14.5, 26)	10.6 ± 9.3	(− 8.7, 35)	0.3200
Full neck length (mm)	100.8 ± 7.0	(88.4, 119)	88 ± 4.7	(78.8, 99)	< 0.0001
Femoral head diameter (mm)	48.4 ± 2.7	(43.6, 53)	41.7 ± 2.4	(37.7, 48)	< 0.0001
D1 (mm)	11.7 ± 4.5	(2.3, 20)	5.8 ± 3.1	(1.5, 15)	< 0.0001
D2 (mm)	6.6 ± 2.9	(1.4, 13)	3.5 ± 2.0	(0.6, 8)	< 0.0001
D3 (mm)	10.3 ± 3.6	(4.3, 18)	6.6 ± 2.4	(2.4, 12)	0.0001
Projection error (%)	19.5 ± 2.7	(15.3, 27)	21.4 ± 4.1	(13.2, 30)	0.0240

*SD* standard deviation

*P*-value = Shapiro-Wilk test *p*-value

D = width of the double contour (including line thickness) at three levels perpendicular to the femoral axis, where D1 lies at the femoral neck level, D3 indicates the maximum thickness of the double line and D2 indicates the minimum thickness between D1 and D3

Intraclass correlation coefficients (ICC) for inter-rater reliability were 0.99, 0.98 and 0.97 for D1, D2 and D3, respectively. For intra-rater reliability, the respective ICCs for D1, D2 and D3 were 0.99, 0.93 and 0.97. Inter-rater reliability for D_H_ and L_N_ were 0.95 and 0.99 respectively.

**Fig. 4:**
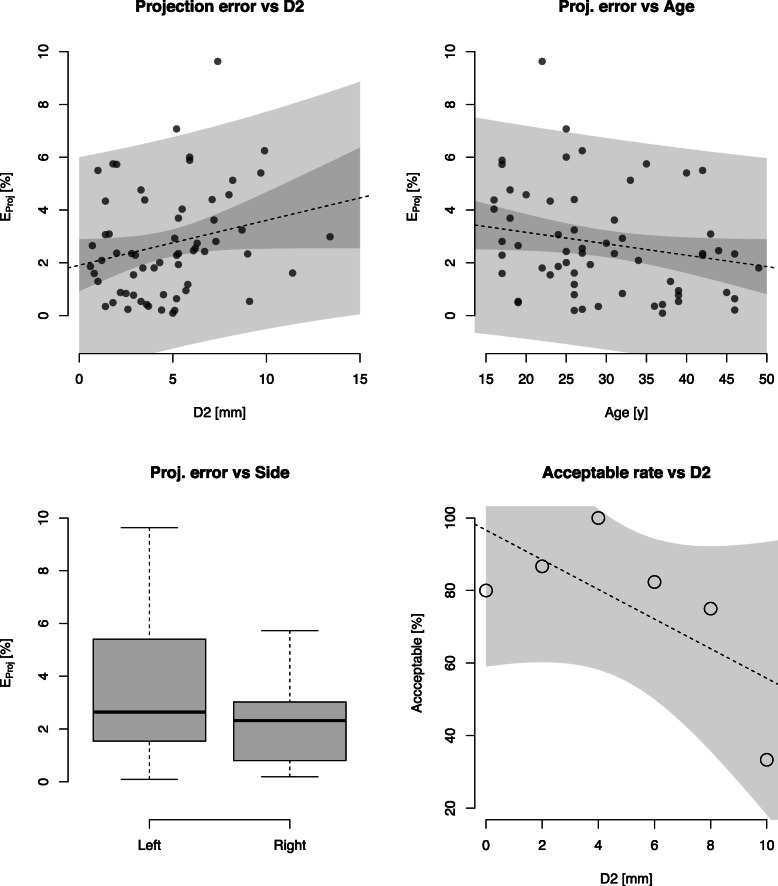
Plots generated with R showing the correlations between (**a**) the D2 double contour width, (**b**) age and (**c**) hip side with projection error. The rate of acceptability of radiographs drops below 80% for D2 widths higher than 5 mm (**d**)

Based on the backward stepwise multivariate regression analysis, antetorsion, gender, D1 and D3 were not associated with projection errors. However, there were associations between projection error and the D2 double contour width (Fig. 4a), age (Fig. 4b) and hip side (right) (Fig. 4c); the respective mean (± standard deviation [SD]) effect sizes were 0.57 ± 0.24 (95% confidence interval [CI]: 0.08, 1.06), − 0.59 ± 0.24 (95%CI: − 1.08, − 0.11), and − 1.06 ± 0.48 (95%CI: − 2.01, − 0.10). For the former, the effect size of 0.57 indicates an increase in projection error of 0.57% for each 1 mm change in D2.

Lastly, the AR was negatively associated with D2 width (− 4% per mm, *p* = 0.02). In particular, AR dropped below 80% for D2 values larger than 5 mm (Fig. 4d).

## Discussion

From our retrospectively analysed imaging data, we wanted to examine whether the double contour created by projection of the greater trochanter posteromedial facet is predictive of the projection error of femoral offset on plain pelvic AP radiographs. Although we found weak correlations between various parameters related to the posteromedial facet and femoral neck projection error, there was a significant trend showing that wider double contours are associated with higher femoral projection errors. Based on our regression modelling, the D2 double contour width—the narrowest width between the contours—correlated with the projection error of femoral offset.

Accurate reconstruction of femoral offset is mandatory in THA. Most surgeons use plain pelvic AP radiographs for templating and postoperative quality control, which often leads to inconsistencies in the correct projection of offset due to inaccurate femoral positioning between the x-ray beam and film. Internal rotation is also often limited in the presence of osteoarthritis, which adds to the difficulties in achieving correct femoral positioning. A reliable predictor for the accuracy of femoral projection on plain pelvic radiographs such as the D2 double contour width would naturally aid the surgeon in daily clinical practice.

When compared to CT scan measurements, radiographic femoral offset is underestimated by over 5 mm (or ~ 15% of neck length) in 28% of THA patients [6]. Based on the fact that a loss of femoral offset by 15% is associated with gait alterations [5], we considered a femoral projection error (leading to an underestimation of femoral offset) of less than 10% as acceptable. Based on this definition, the proportion of acceptable radiographs dropped below 80% when D2 was wider than 5 mm. The D1 (width at the level of the femoral neck) and D3 (width at the level of the piriformis fossa) double contour widths were less reliable predictors of femoral malrotation; this might be explained by the variable shape of the double contour, which could affect D1 and D3 to a greater extent than D2.

Being female and younger were associated with a higher offset error. We speculate that for these patients who are generally smaller, the measurement errors are proportionally larger. We also observed a small but significant difference in offset error between right and left hips. This fits with findings from Sutter et al who reported higher antetorsion in the left legs of both healthy and femoroacetabular impingement patients [15]. This asymmetry could potentially favour projection errors on one side since patient positioning protocols are not side-specific. Assessment of bilateral imaging in large cohorts would be needed to confirm or reject any of these conjectures.

Interestingly, there was no statistical association between antetorsion and projection error, although in theory, under perfectly standardized patient positioning, this association should exist. This reflects how perfect positioning is hard to achieve in real life, and further justify the need for a simple projection quality control procedure.

The inter- and intra-rater reliability of our double contour width measurements were very high. All examiners used the same assessment protocol, which provided a clear guideline instructing them on how to evaluate the double contour. We believe the evaluation of the double contour is easy and reproducible, and recommend its’ use in assessing the reliability of a radiograph before measuring femoral offset.

There are several limitations of our study. The study design is retrospective in nature and its power is limited only to patients with a medical indication (most likely affecting the hip joint) who required MRI and radiograph assessments. Most, if not all, of our cohort included patients with hip problems, especially at a young age. We cannot consider our patients as healthy candidates regarding joint function, since many had an anatomical issue as the cause of their dysfunction; our study cohort is not representative of the general population. There can be anatomical variants that do not present the associations observed in our cohort, but a much larger cohort would be needed to identify these variants. The regression parameters would differ with another patient cohort, nonetheless, we assume our results are still highly relevant for THA patients, since we selected patients who consulted for hip problems. Further evaluation of the double contour in a cohort without hip disorders as an indication for the acquisition of an MRI/CT and radiograph would be helpful to test our study hypothesis in a healthy population.

## Conclusion

Our data indicate that if the minimal width of the trochanteric double contour between the level of the lateral femoral neck and piriformis fossa exceeds 5 mm on radiographs from patients with a hip disorder, the risk of underestimating femoral offset by more than 10% is significantly greater compared to narrower double contours. On this basis, the trochanteric double contour is a potential landmark for excluding pelvic AP radiographs unsuitable for THA templating. Using this particular landmark could greatly assist lower extremity surgeons in discerning between those radiographs that are suitable or not during the routine preparation for THA.

## Data Availability

The datasets during and/or analyzed during the current study are available from the corresponding author on reasonable request.

## Abbreviations

AP: Anteroposterior
AR: Acceptability rate
CI: Confidence interval
CT: Computed tomography
D: Double contour width (where, for example, D1 = double contour width at femoral neck level)
D_H_: True femoral head diameter
D_H_*: Apparent femoral head diameter
ICC: Intraclass correlation coefficients
L_N_: True femoral neck axis length
L_N_*: Apparent femoral neck axis length
MRI: Magnetic resonance image
SD: Standard deviation
THA: Total hip arthroplasty
